# Puerarin Attenuates Osteoarthritis via Multi‐Target Regulation of Inflammation, Apoptosis, and ECM Degradation

**DOI:** 10.1111/jcmm.70833

**Published:** 2025-09-25

**Authors:** Yanjing Zhang, Zhiqiang Chen, Ye Cheng, Yuxuan Zhou, Yaoheng Yang, Mengjiao Che, Yiwen Zhang, Yalan Li

**Affiliations:** ^1^ Department of Anesthesiology, The Eighth Affiliated Hospital Southern Medical University (The First People's Hospital of Shunde, Foshan) Foshan City China; ^2^ Department of Anesthesiology The First Affiliated Hospital of Jinan University Guangzhou China

**Keywords:** apoptosis, bioinformatics, chondrocytes, extracellular matrix, inflammation, osteoarthritis, PI3K‐AKT signalling pathway, Puerarin

## Abstract

Osteoarthritis (OA) is a degenerative joint disease characterised by cartilage breakdown, leading to pain and reduced quality of life. This study aims to investigate the therapeutic potential of Puerarin (PUE), a natural compound derived from *Pueraria lobata* , in modulating OA progression. Employing a multifaceted approach that included bioinformatics analysis, molecular docking, in vitro assays, and in vivo experiments, we identified 57 overlapping targets between PUE and OA‐related genes, suggesting a multi‐target interaction model. Our findings revealed that PUE effectively inhibited inflammatory cytokine production and protected chondrocytes from apoptosis at non‐cytotoxic concentrations (5 and 10 μM), promoting extracellular matrix synthesis by reversing IL‐1β‐induced degradation of Aggrecan and Collagen II while reducing MMP‐13 and ADAMTS5 expression. Furthermore, PUE was shown to attenuate IL‐1β‐induced apoptosis by restoring BCL‐2 levels and decreasing cleaved caspase‐3 levels. In vivo, PUE administration in a destabilisation of the medial meniscus (DMM) mouse model significantly slowed OA progression, preserving cartilage structure and reducing osteophyte formation. Moreover, PUE activated the PI3K‐AKT signalling pathway, underscoring its anti‐inflammatory and anti‐apoptotic mechanisms. Collectively, these results support Puerarin's potential as a disease‐modifying agent in OA treatment, warranting further clinical exploration and consideration of its application in combination therapies to enhance cartilage protection and repair.

## 1 Introduction

Osteoarthritis (OA) is a prevalent degenerative joint disease characterised by the progressive breakdown of articular cartilage, resulting in pain, stiffness, and functional impairment, which significantly diminish the quality of life for affected individuals [1, 2, 3]. It is associated with various risk factors, including age, obesity, and previous joint injuries, leading to substantial economic burdens on healthcare systems worldwide [4, 5, 6]. Current treatment modalities primarily focus on symptomatic relief through analgesics and anti‐inflammatory agents, yet they fail to address the underlying mechanisms of cartilage degeneration and disease progression [7, 8]. This limitation underscores the urgent need for novel therapeutic strategies that not only alleviate symptoms but also modify the course of OA.

Recent studies have highlighted the potential of natural compounds with anti‐inflammatory and chondroprotective properties in managing OA [9, 10]. One such candidate is Puerarin (PUE), an isoflavonoid extracted from the traditional Chinese medicinal herb *Pueraria lobata* . PUE has exhibited a range of beneficial biological activities, including anti‐inflammatory effects and enhancement of cartilage health [11]. Previous investigations have demonstrated that PUE can inhibit key inflammatory mediators and promote chondrocyte survival, suggesting its relevance as a therapeutic agent for OA management [12]. However, a comprehensive understanding of its molecular mechanisms in the context of OA remains incomplete, necessitating further exploration.

To address this gap, our study employs an integrative approach combining bioinformatics analysis, molecular docking, in vitro assays, and in vivo experimentation to elucidate the effects of PUE on OA. Utilising tools like the SwissTargetPrediction database, we first identified potential target proteins associated with PUE and cross‐referenced these with OA‐related genes. This systematic analysis revealed a network of overlapping targets that may mediate the therapeutic effects of PUE in OA, indicating a multi‐target interaction strategy [13]. Additionally, we performed cell viability assays to determine non‐cytotoxic concentrations of PUE for chondrocyte treatments, ensuring the safety and efficacy of subsequent experiments.

The primary objective of this research is to delineate the protective effects of PUE on chondrocytes, specifically focusing on its role in modulating inflammatory responses and apoptosis in the context of OA. By investigating PUE's potential to preserve extracellular matrix (ECM) integrity and promote chondrocyte survival, we aim to establish its viability as a disease‐modifying agent for OA [7]. Furthermore, this study will explore the underlying molecular pathways involved, particularly those associated with inflammation and apoptosis, thereby contributing to the development of more effective treatment strategies for OA.

In conclusion, as the prevalence of osteoarthritis continues to rise, the exploration of novel therapeutic agents such as PUE presents an exciting opportunity to not only manage symptoms but also potentially alter the disease trajectory. Through our multifaceted approach, we hope to provide valuable insights into PUE's therapeutic potential and pave the way for future clinical applications in the management of osteoarthritis.

## 2 Materials and Methods

### 2.1 Bioinformatic Prediction

The chemical structure of PUE was retrieved from the PubChem database. Target prediction was performed using the SwissTargetPrediction database. Osteoarthritis (OA)‐related genes were identified from the GeneCards database. A compound‐target‐disease network was constructed using Cytoscape software. Protein–protein interaction (PPI) networks were generated using the STRING database. Gene Ontology (GO) and Kyoto Encyclopaedia of Genes and Genomes (KEGG) enrichment analyses were conducted using R software.

### 2.2 Molecular Docking

Molecular docking was performed using established protocols. AutoDock software was used to simulate docking between PUE (PubChem CID: 5281807) and PI3K (PDB ID: 4OVU) or AKT (PDB ID: 4EJN). Structural preprocessing was conducted in PyMOL version 2.6.0. The Lamarckian genetic algorithm was applied, with the key residues of the binding pocket kept rigid. Docking involved 2.5 million energy evaluations, and the optimal binding conformation was selected based on cluster analysis.

### 2.3 Chondrocyte Isolation and Culture

Articular cartilage was harvested from the knee joints of 4‐week‐old C57BL/6 mice euthanised via intraperitoneal overdose of pentobarbital. Cartilage was minced into ~1 mm^3^ fragments and digested in 2 mg/mL type II collagenase at 37°C for 4 h. After centrifugation, cells were resuspended in DMEM/F12 medium supplemented with 10% fetal bovine serum (FBS) and 1% penicillin/streptomycin and incubated at 37°C in a 5% CO_2_ atmosphere. Cells were passaged using 0.25% trypsin once 80%–90% confluence was reached. Cells at passages 1–3 were used for experiments to ensure phenotypic stability.

### 2.4 Cell Viability and Cytotoxicity Assay

Puerarin (Catalogue No. P5555, Merck KGaA, Darmstadt, Germany) was tested for cytotoxicity using a CCK‐8 assay. Chondrocytes (4 × 10^3^ cells/well) were seeded in 96‐well plates and treated with various concentrations of PUE. At 24, 48, and 72 h, 10 μL CCK‐8 solution was added and incubated for 2 h. Absorbance was measured at 450 nm.

### 2.5 Toluidine Blue Staining

After removing the medium and washing cells twice with PBS (1 min each), toluidine blue solution (Solarbio) was added for 15 min. Cells were washed three times with deionised water and observed under a light microscope.

### 2.6 Safranin O Staining

Cells were rinsed twice with PBS (1 min each), stained with Safranin O solution (Solarbio) for 30 min, washed with deionised water, and observed microscopically.

### 2.7 Immunofluorescence Staining

Chondrocytes were seeded on glass slides and treated with IL‐1β and PUE. After fixation with 4% paraformaldehyde for 20 min and permeabilisation with 0.5% Triton X‐100 for 10 min, cells were blocked with 10% goat serum for 1 h. Primary antibodies (Collagen II, Aggrecan, ADAMTS5, MMP13; all at 1:200) were applied and incubated overnight at 4°C. Secondary antibodies conjugated with Alexa Fluor 488 or 594 (1:500) were added and incubated for 2 h at 37°C. Fluorescence was visualised using a fluorescence microscope.

### 2.8 Inflammatory Cytokine Assays

Chondrocytes were treated with 10 ng/mL IL‐1β and various concentrations of PUE for 24 h [14]. Nitric oxide (NO) levels were measured using the Griess reagent at 540 nm. The concentrations of PGE2, TNF‐α, and IL‐6 were quantified using ELISA kits. All experiments were performed in triplicate.

### 2.9 TUNEL Apoptosis Assay

Apoptosis was assessed using the TUNEL assay kit (Beyotime). Cells were fixed with 4% paraformaldehyde for 20 min and permeabilised with 0.3% Triton X‐100 for 5 min. A TUNEL reaction mixture (TdT enzyme: label solution = 1:9) was applied at 50 μL per well and incubated at 37°C in the dark for 1 h. After mounting with DAPI‐containing medium, apoptotic cells were visualised by fluorescence microscopy. The apoptosis rate was quantified using ImageJ.

### 2.10 RNA Extraction and qRT‐PCR

Total RNA was extracted using TRIzol reagent and quantified by Nanodrop spectrophotometry. cDNA was synthesised using reverse transcription, and qRT‐PCR was conducted using SYBR Green reagents on a LightCycler 96 System (Roche). Relative expression levels were calculated using the 2^−ΔΔCt^ method. Primer sequences are listed in Table 1.

**TABLE 1:** Sequences of all primers used in qPCR.

Gene	Forward primer (5′→3′)	Reverse primer (5′→3′)
iNOS	CATTGGAAGTGAAGCGTTTCG	CAGCTGGGCTGTACAAACCTT
COX‐2	ACACACTCTATCACTGGCACC	TTCAGGGAGAAGCGTTTGC
TNF‐α	CCTCTTCTCATTCCTGCTTGTGG	GGCCATTTGGGAACTTCTCATC
IL‐6	TGTTCTCTGGGAAATCGTGGAA	GCAAGTGCATCATCGTTGTTCA

### 2.11 Western Blot Analysis

Total proteins were extracted using RIPA buffer and quantified via BCA assay. Equal amounts of protein (20 μg) were separated by 10% SDS‐PAGE and transferred to PVDF membranes. After blocking with 5% non‐fat milk, membranes were incubated overnight with primary antibodies and subsequently with HRP‐conjugated secondary antibodies. Protein bands were detected using ECL reagents and imaged with a Bio‐Rad ChemiDoc XRS+ system. Image Lab V3.0 software was used for analysis. Antibodies used included Collagen II, ADAMTS5, MMP13, BAX (Abcam); Cleaved caspase‐3, PI3K, p‐PI3K, AKT, p‐AKT, mTOR (CST); and BCL‐2, β‐actin (Proteintech).

### 2.12 Animal Studies

A total of 32 male C57BL/6 mice (8 weeks old) were used to establish osteoarthritis (OA) models via destabilisation of the medial meniscus (DMM). Mice were randomly assigned to four groups: Control, DMM, DMM + Puerarin (PUE, 50 mg/kg), and DMM + PUE (100 mg/kg). In the Control group, mice underwent skin incision and joint capsule opening without DMM, ensuring methodological consistency with the DMM group while avoiding surgical induction of OA. PUE was administered by oral gavage three times per week for 8 weeks. All animal procedures were approved by the Institutional Animal Care and Use Committee (IACUC) of the Third Affiliated Hospital of Southern Medical University under protocol number TOP‐IACUC‐2023‐0333. The animals were sourced from Shenzhen Tongqiao Biotechnology Co. Ltd. (Shenzhen, China). This study was conducted in compliance with the ARRIVE 2.0 guidelines (https://arriveguidelines.org), ensuring ethical standards and reproducibility.

### 2.13 Micro‐CT

Micro‐CT scanning was performed using the SkyScan‐1276 system (Bruker) at a resolution of 9 μm/pixel to evaluate subchondral bone structure of the tibial plateau. Parameters such as bone volume/tissue volume (BV/TV) were analysed.

### 2.14 Histological Analysis: H&E and SO/FG Staining

After decalcification and paraffin embedding, knee joint sections were stained with haematoxylin and eosin (H&E) and Safranin O/Fast Green (SO/FG) to assess cartilage structure. Cartilage degeneration was evaluated using the OARSI scoring system [15]. Three independent observers, blinded to the experimental groups, assessed the histological sections separately. The final OARSI score for each sample was calculated as the mean value of the three observers' scores.

### 2.15 Immunohistochemistry and Immunofluorescence in Tissues

Immunohistochemistry was performed to detect Aggrecan expression in joint cartilage sections. TUNEL staining was applied to evaluate chondrocyte apoptosis in tissue samples.

### 2.16 Statistical Analysis

All experiments were performed at least in triplicate. Data are presented as mean ± standard deviation (SD). Statistical comparisons between two groups were performed using two‐tailed Student's *t*‐test, while comparisons among multiple groups were analysed using one‐way ANOVA followed by Bonferroni's post hoc test. Statistical significance was set at *p < 0.05, **p < 0.01, and ***p < 0.001. GraphPad Prism 9.0 software was used for all analyses.

## 3 Result

### 3.1 Bioinformatics Analysis of PUE in Osteoarthritis

**FIGURE 1:**
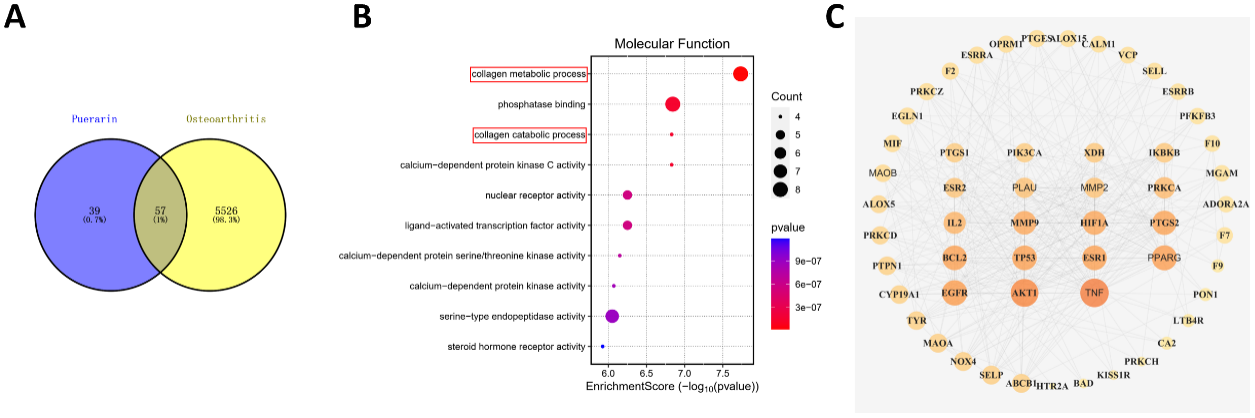
Bioinformatics analysis of potential Puerarin targets in osteoarthritis. (A) Venn diagram showing the overlap between predicted Puerarin targets (*n* = 39) obtained from SwissTargetPrediction and osteoarthritis‐related genes (*n* = 5526) from the GeneCards database, identifying 57 common targets. (B) Gene Ontology (GO) enrichment analysis of the overlapping targets, highlighting significant biological processes related to collagen metabolism, extracellular matrix organisation, and connective tissue structure. (C) Protein–protein interaction (PPI) network constructed from the 57 shared targets, with key hub genes including BCL2, PTGS2, MMP9, HIF1A, and AKT1 identified as central nodes potentially involved in OA‐related inflammation and matrix remodelling.

To investigate the potential molecular mechanisms by which PUE may exert therapeutic effects in osteoarthritis (OA), we first used the SwissTargetPrediction database to visualise the chemical structure of PUE and predict its associated targets, resulting in 39 potential target proteins (Figure 1A). Simultaneously, 5526 OA‐related genes were identified using the GeneCard database. Cross‐referencing these datasets yielded 57 overlapping targets, which may represent the potential functional mediators of PUE in OA treatment (Figure 1A). Subsequent Gene Ontology (GO) enrichment analysis revealed that these targets were significantly enriched in biological processes related to collagen metabolic and catabolic processes, ECM organisation, and connective tissue structural integrity—key elements implicated in OA pathogenesis (Figure 1B). To further explore the functional interconnectivity of these targets, a protein–protein interaction (PPI) network was constructed. Central nodes in this network included BCL2, PTGS2, MMP9, HIF1A, and AKT1, indicating their potential roles as core regulatory proteins involved in inflammation, apoptosis, and matrix remodelling in the OA microenvironment (Figure 1C). Collectively, these bioinformatics analyses suggest that PUE may modulate OA progression through multi‐target interactions, particularly influencing pathways related to ECM degradation and inflammatory signalling.

### 3.2 Optimal Concentration of PUE on Chondrocytes

**FIGURE 2:**
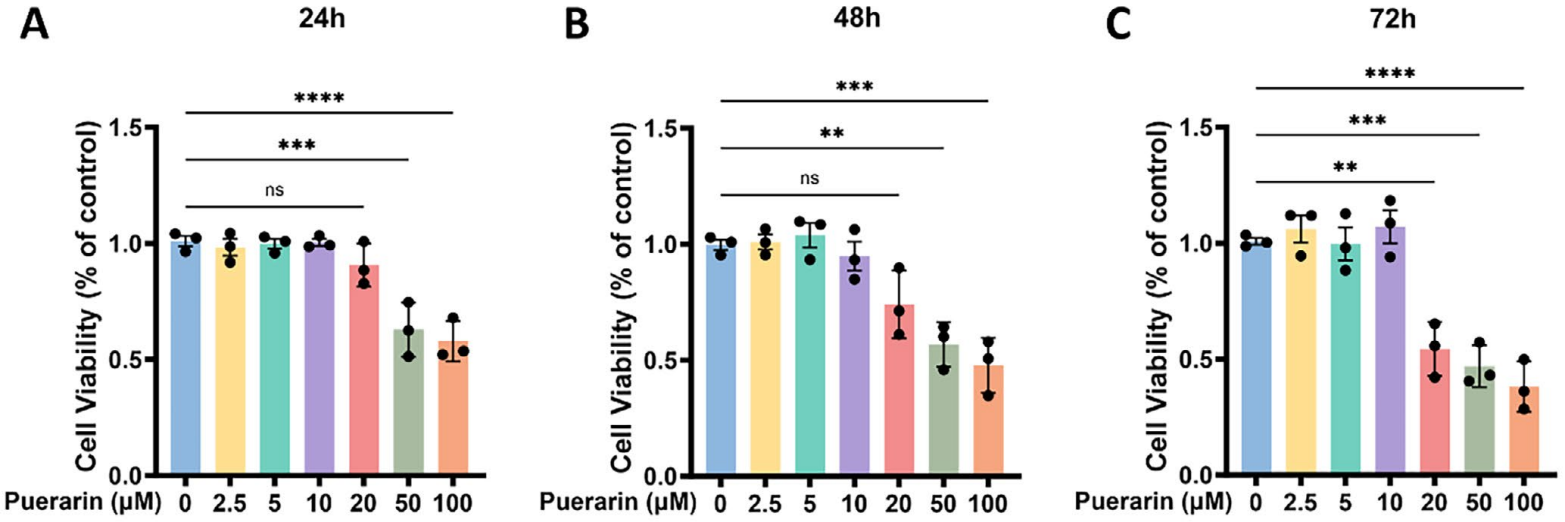
CCK‐8 assay of chondrocyte viability following Puerarin treatment at different time points. (A) Chondrocyte viability assessed by CCK‐8 assay after 24‐h treatment with Puerarin at concentrations of 0, 2.5, 5, 10, 20, 50, and 100 μM. (B) CCK‐8 assay results for chondrocytes treated with the same concentrations of Puerarin for 48 h. (C) CCK‐8 assay analysis of chondrocyte viability following 72‐hour treatment with increasing concentrations of Puerarin. Statistical significance was set at *p < 0.05, **p < 0.01, ***p < 0.001 and ****p < 0.0001.

To identify a non‐cytotoxic concentration range of PUE for chondrocyte‐based assays, cell viability was evaluated using the CCK‐8 assay across a series of concentrations (0, 2.5, 5, 10, 20, 50, and 100 μM) at 24, 48, and 72 h. The results demonstrated that at 24 and 48 h, PUE at 50 and 100 μM significantly decreased chondrocyte viability (*p* < 0.05), whereas lower concentrations up to 20 μM did not exhibit notable cytotoxicity (Figure 2A,B). Interestingly, at the 72‐h time point, PUE at 20 μM also began to exhibit significant cytotoxic effects, in addition to the continued suppression observed at 50 and 100 μM (*p* < 0.05), while concentrations of 2.5, 5, and 10 μM remained well tolerated (Figure 2C). Based on these findings, 5 and 10 μM were selected as the optimal concentrations for subsequent in vitro experiments, ensuring minimal cytotoxicity while maintaining potential biological efficacy.

### 3.3 Puerarin Suppresses IL‐1β‐Induced ECM Degradation

**FIGURE 3:**
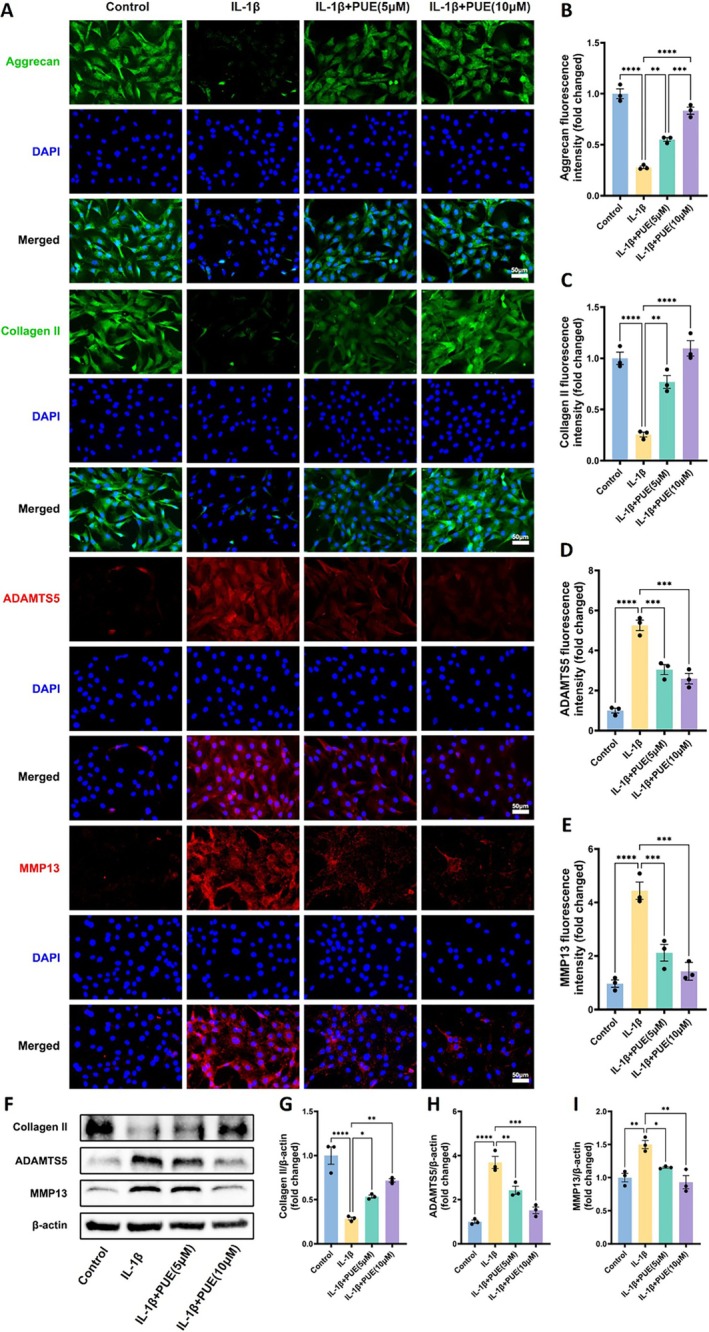
Immunofluorescence and Western blot analysis of extracellular matrix components in IL‐1β–stimulated chondrocytes following Puerarin treatment. (A) Representative immunofluorescence images showing the expression of Aggrecan, Collagen II, ADAMTS5, and MMP13 in chondrocytes under different treatment conditions (Control, IL‐1β, IL‐1β + PUE 5 μM, IL‐1β + PUE 10 μM). Nuclei were counterstained with DAPI (blue). Scale bars, 50 μm. (B–E) Quantification of fluorescence intensity (fold change) for Aggrecan (B), Collagen II (C), ADAMTS5 (D), and MMP13 (E). (F) Western blot analysis of Collagen II, ADAMTS5, and MMP13 protein levels, with β‐actin as the loading control. (G–I) Densitometric quantification of protein expression normalised to β‐actin for Collagen II (G), ADAMTS5 (H), and MMP13 (I). Data are presented as mean ± SD. Statistical significance was set at *p < 0.05, **p < 0.01, ***p < 0.001, and ****p < 0.0001.

Extracellular matrix degradation is a hallmark of osteoarthritis (OA) progression, primarily characterised by the loss of key matrix components. To evaluate the protective role of PUE against IL‐1β–induced ECM breakdown, immunofluorescence staining was performed to assess the expression of Aggrecan, Collagen II, ADAMTS5, and MMP‐13. As shown in Figure 3A–E, IL‐1β stimulation markedly reduced the fluorescence intensity of Aggrecan and Collagen II, indicating suppression of ECM synthesis, while significantly enhancing the expression of the matrix‐degrading enzymes MMP‐13 and ADAMTS5. In contrast, treatment with PUE at both 5 and 10 μM concentrations effectively reversed these changes, as evidenced by increased levels of Aggrecan and Collagen II, and decreased levels of ADAMTS5 and MMP‐13, suggesting a protective role in maintaining ECM integrity. These findings were further corroborated by Western blot analysis (Figure 3F–I), which demonstrated consistent upregulation of Collagen II and downregulation of ADAMTS5 and MMP‐13 following PUE intervention. Collectively, these results indicate that PUE promotes ECM synthesis and inhibits its degradation under inflammatory conditions, highlighting its potential as a therapeutic agent in OA management.

### 3.4 Puerarin Attenuates IL‐1β–Induced Chondrocyte Apoptosis

**FIGURE 4:**
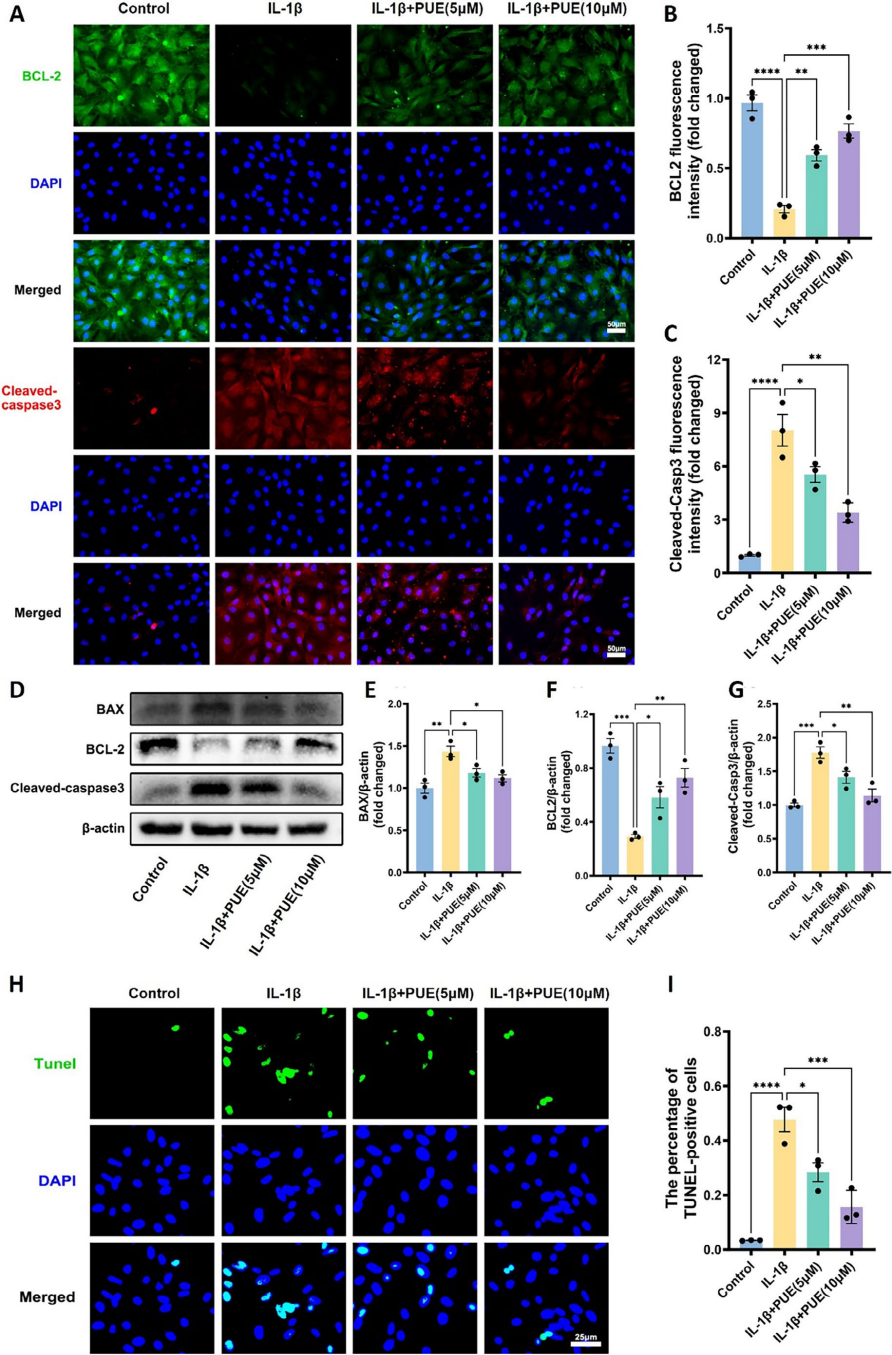
Effects of Puerarin on IL‐1β–induced apoptosis in chondrocytes. (A) Representative immunofluorescence images of BCL‐2 and cleaved caspase‐3 in chondrocytes under different treatment conditions (Control, IL‐1β, IL‐1β + PUE 5 μM, IL‐1β + PUE 10 μM). Nuclei were counterstained with DAPI (blue). Scale bars, 50 μm. (B, C) Quantification of fluorescence intensity (fold change) for BCL‐2 (B) and cleaved caspase‐3 (C). (D) Western blot analysis of BAX, BCL‐2, and cleaved caspase‐3, with β‐actin as the loading control. (E–G) Densitometric quantification of Western blot bands for BAX (E), BCL‐2 (F), and cleaved caspase‐3 (G), normalised to β‐actin. (H) TUNEL staining of chondrocytes under indicated treatments to assess apoptotic cell populations. Nuclei were stained with DAPI (blue). Scale bars, 25 μm. (I) Quantification of TUNEL‐positive cells (percentage of total cells). Data are presented as mean ± SD. Statistical significance was set at *p < 0.05, **p < 0.01, ***p < 0.001, and ****p < 0.0001.

To assess the anti‐apoptotic effects of PUE in IL‐1β–stimulated chondrocytes, we first evaluated the expression of apoptosis‐related markers using immunofluorescence staining. As shown in Figure 4A–C, IL‐1β significantly downregulated the expression of the anti‐apoptotic protein BCL‐2 and upregulated cleaved caspase‐3 levels, indicative of increased apoptosis. However, treatment with PUE effectively reversed these changes in a dose‐dependent manner, restoring BCL‐2 levels and reducing cleaved caspase‐3 expression. Western blot analysis further confirmed these findings (Figure 4D–G), revealing that IL‐1β enhanced the expression of pro‐apoptotic proteins BAX and cleaved caspase‐3 while suppressing BCL‐2. PUE treatment mitigated these effects by downregulating BAX and cleaved caspase‐3 and upregulating BCL‐2 in a concentration‐dependent fashion. Complementary to these molecular findings, TUNEL staining (Figure 4H,I) demonstrated a significantly higher percentage of apoptotic cells in the IL‐1β‐treated group, which was markedly reduced following PUE intervention. These data collectively suggest that PUE can protect chondrocytes from IL‐1β–induced apoptosis by modulating key regulators of the intrinsic apoptotic pathway, thereby contributing to its chondroprotective effects in osteoarthritis.

### 3.5 Puerarin Modulates IL‐1β‐Induced Inflammatory Response

**FIGURE 5:**
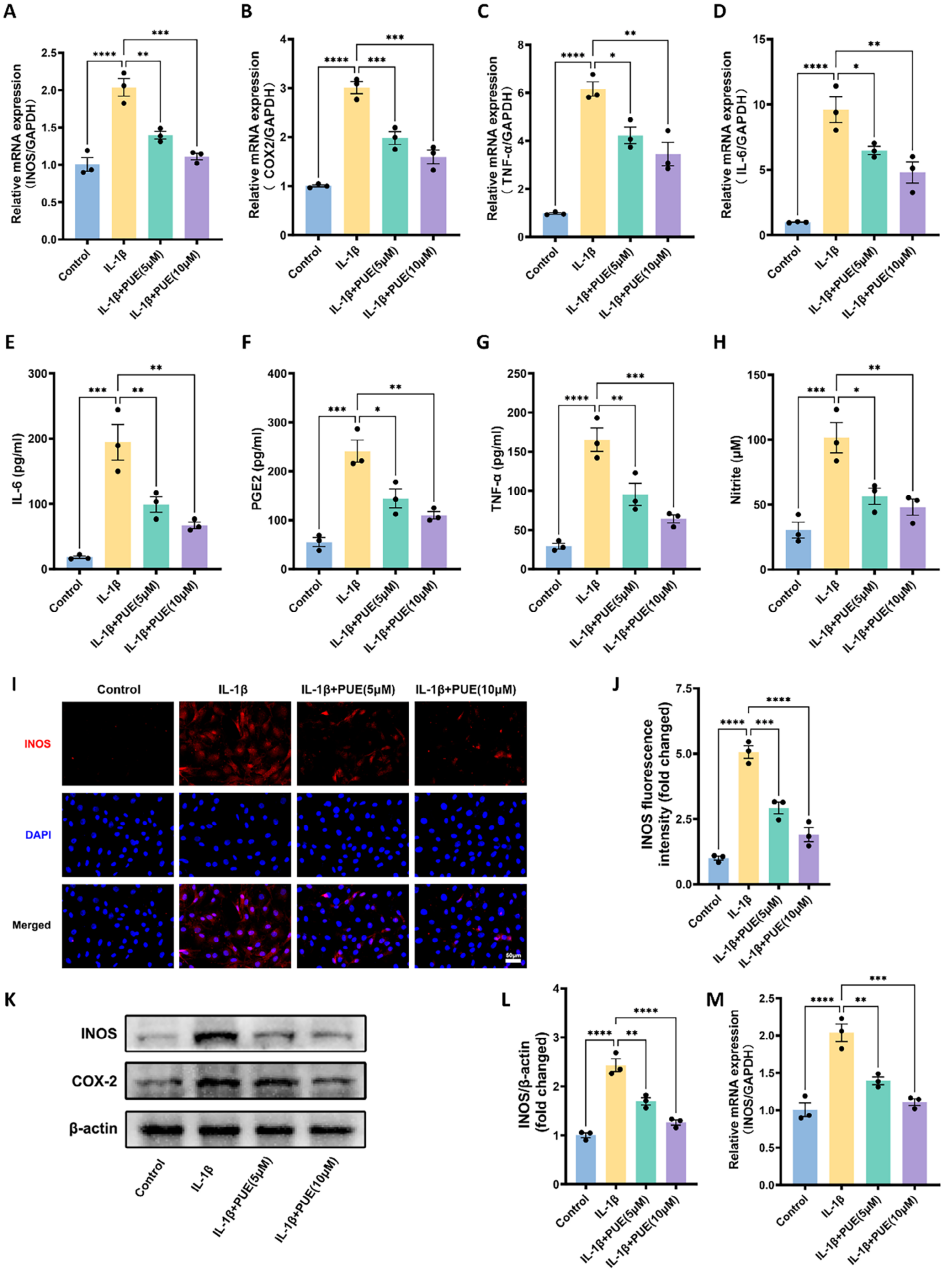
Effects of Puerarin on inflammatory mediator expression in IL‐1β–stimulated chondrocytes. (A–D) Relative mRNA expression levels of iNOS (A), COX‐2 (B), TNF‐α (C), and IL‐6 (D) measured by quantitative real‐time PCR in chondrocytes treated with IL‐1β and/or Puerarin (5 or 10 μM). GAPDH was used as the internal control. (E–H) ELISA quantification of IL‐6 (E), PGE2 (F), TNF‐α (G), and nitrite (H) levels in the supernatants of chondrocyte cultures under the indicated treatment conditions. (I) Representative immunofluorescence images showing iNOS expression in chondrocytes under the indicated treatments. (J) Quantitative fluorescence intensity analysis of iNOS expression. (K) Western blot analysis of iNOS and COX‐2 protein expression in chondrocytes under the indicated treatments. (L) Densitometric quantification of iNOS protein expression. (M) Densitometric quantification of COX‐2 protein expression. Data are presented as mean ± SD. Statistical significance was set at *p < 0.05, **p < 0.01, ***p < 0.001, and ****p < 0.0001.

Inflammation plays a central role in ECM degradation during osteoarthritis (OA) progression. To further explore the anti‐inflammatory potential of PUE, we assessed its effects on key inflammatory mediators including TNF‐α, IL‐6, iNOS, and COX‐2. As shown in Figure 5A–D, quantitative real‐time PCR analysis revealed that IL‐1β significantly upregulated the mRNA expression of these inflammatory genes (*p* < 0.05), while co‐treatment with PUE notably suppressed their expression in a dose‐dependent manner, with 10 μM showing greater efficacy than 5 μM. Consistently, ELISA results demonstrated that IL‐1β markedly increased the secretion of IL‐6, TNF‐α, prostaglandin E2 (PGE2), and nitrite, all of which were significantly reduced by PUE treatment (Figure 5E–H). Moreover, immunofluorescence staining further confirmed that IL‐1β robustly enhanced iNOS expression in chondrocytes, whereas PUE treatment markedly attenuated this increase (Figure 5I). Quantitative fluorescence analysis (Figure 5J) supported these observations, showing a significant reduction in iNOS intensity following PUE administration. In line with these findings, Western blot assays demonstrated that IL‐1β strongly induced the protein expression of iNOS and COX‐2, which was dose‐dependently suppressed by PUE (Figure 5K). Densitometric analysis revealed significant downregulation of both iNOS (Figure 5L) and COX‐2 (Figure 5M) protein levels, particularly at 10 μM PUE. These findings strongly suggest that PUE attenuates the inflammatory response in IL‐1β–stimulated chondrocytes by downregulating pro‐inflammatory cytokine production and associated signalling molecules, thereby potentially mitigating ECM degradation and cartilage damage in OA.

### 3.6 Molecular Docking and Regulatory Effects of PUE on the PI3K‐AKT Pathway

**FIGURE 6:**
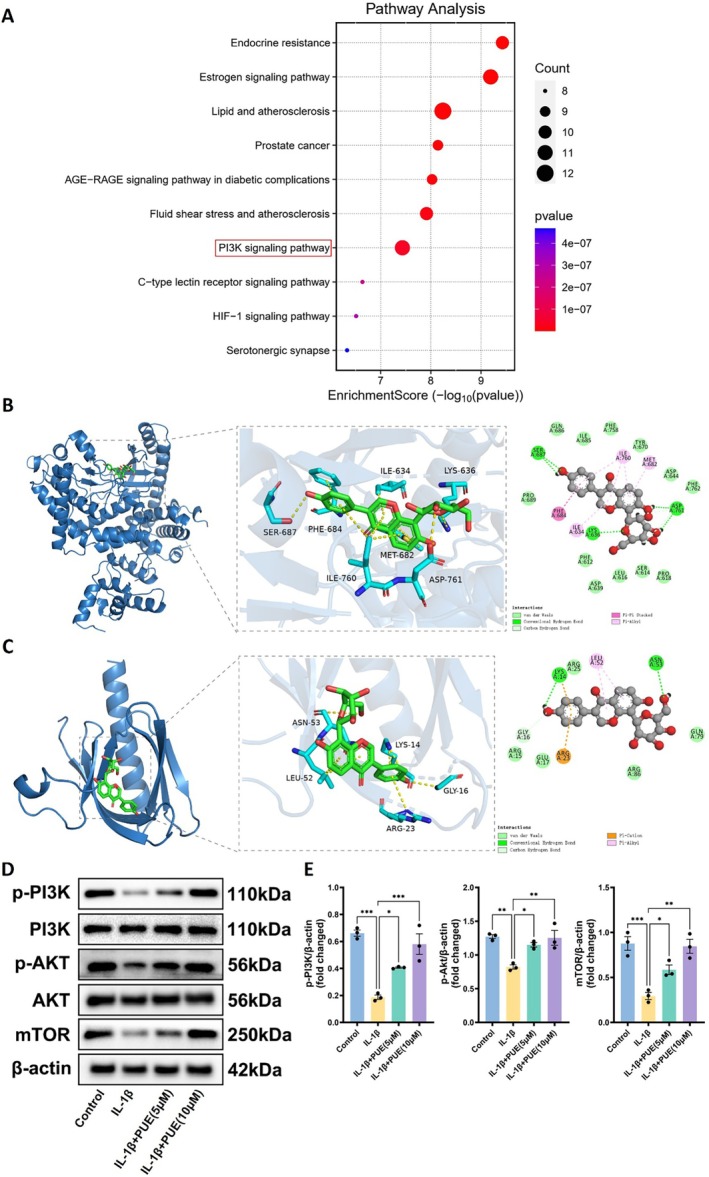
Puerarin modulates the PI3K‐AKT signalling pathway in IL‐1β–stimulated chondrocytes. (A) KEGG pathway enrichment analysis of predicted Puerarin targets, with the PI3K signalling pathway highlighted among the top enriched pathways. (B, C) Molecular docking analysis showing the binding interactions of Puerarin with PI3K (B) and AKT (C). Key hydrogen bonds and hydrophobic interactions between Puerarin and active site residues are displayed in the enlarged panels. (D) Western blot analysis of PI3K, p‐PI3K, AKT, p‐AKT, and mTOR expression levels in chondrocytes under the indicated treatments. β‐actin was used as the loading control. (E) Quantification of p‐PI3K, p‐AKT, and mTOR protein levels normalised to β‐actin. Data are presented as mean ± SD. Statistical significance was set at *p < 0.05, **p < 0.01, ***p < 0.001.

To further elucidate the molecular mechanisms underlying the chondroprotective effects of PUE, we conducted KEGG pathway enrichment analysis based on potential targets identified in earlier bioinformatics screening. The analysis highlighted the PI3K‐Akt signalling pathway as one of the most significantly enriched pathways (Figure 6A), suggesting a central role in mediating PUE's biological effects. Molecular docking analysis demonstrated strong binding affinities between PUE and key proteins in the pathway, including PI3K and AKT. Specifically, PUE formed multiple stable hydrogen bonds and hydrophobic interactions with active site residues of PI3K (ILE‐634, MET‐682, ASP‐761) and AKT (LYS‐14, ARG‐23, GLY‐16), indicating high docking compatibility and potential regulatory interactions (Figure 6B,C). To validate the functional consequences of these interactions, Western blot analysis was performed. As shown in Figure 6D,E, IL‐1β stimulation significantly downregulated the phosphorylated forms of PI3K, AKT, and mTOR, whereas total PI3K and AKT levels remained unchanged. Notably, treatment with PUE dose‐dependently restored phosphorylation levels of PI3K and AKT and enhanced mTOR expression, suggesting reactivation of the PI3K‐Akt–mTOR signalling axis. These results collectively indicate that PUE may exert its anti‐inflammatory and anti‐apoptotic effects in osteoarthritis by directly binding to and modulating components of the PI3K‐AKT pathway.

### 3.7 Puerarin Delays Osteoarthritis Progression in a DMM‐Induced Murine Model

**FIGURE 7:**
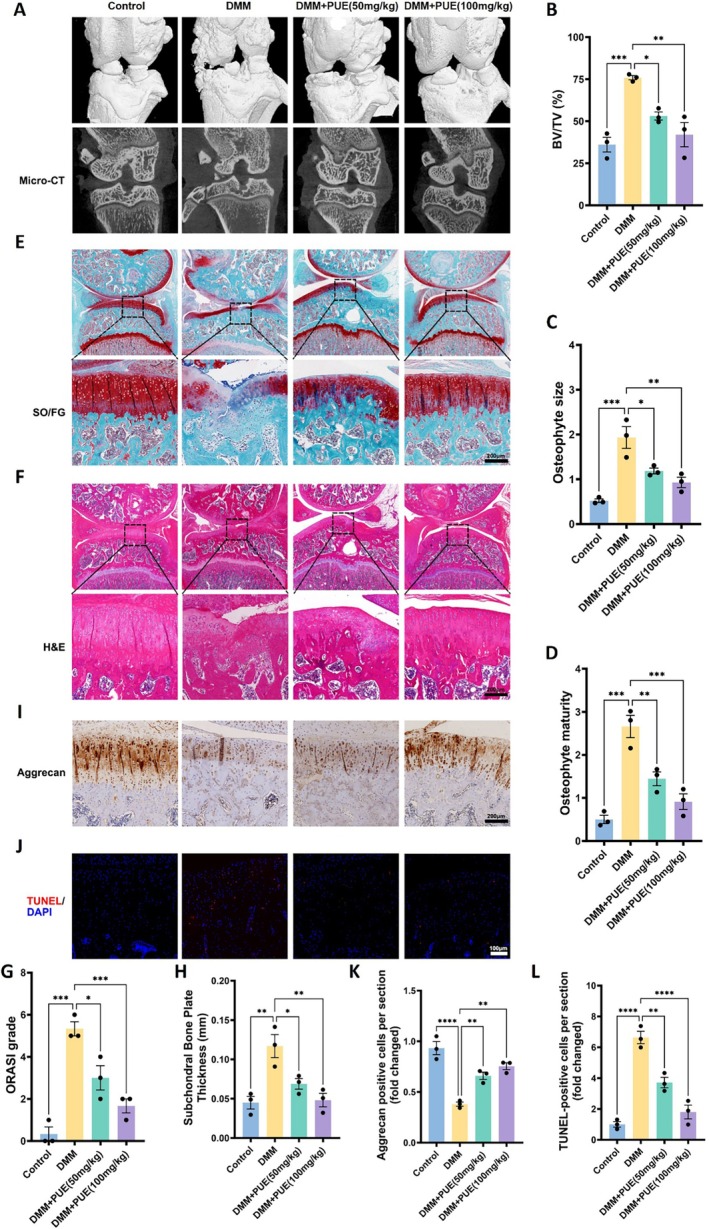
Puerarin mitigates osteoarthritic changes in a DMM‐induced murine model of osteoarthritis. (A) Representative 3D reconstructions and sagittal micro‐CT images of knee joints from each treatment group (Control, DMM, DMM + PUE 50 mg/kg, DMM + PUE 100 mg/kg). (B) Quantification of bone volume/tissue volume ratio (BV/TV, %). (C, D) Quantification of osteophyte size (C) and osteophyte maturity scores (D). (E, F) Representative histological staining of knee joints with Safranin O/Fast Green (SO/FG) (E) and Haematoxylin and Eosin (H&E) (F), showing cartilage and joint structure. Scale bars, 200 μm. (G, H) Quantification of OARSI scores (G) and subchondral bone plate thickness (H). (I, K) Representative images (I) and quantification (K) of Aggrecan immunohistochemical staining in articular cartilage. Scale bars, 200 μm. (J, L) TUNEL staining (J) and quantification (L) of apoptotic cells in articular cartilage sections. Scale bars, 100 μm. Data are presented as mean ± SD. Statistical significance was set at *p < 0.05, **p < 0.01, ***p < 0.001, and ****p < 0.0001.

In the in vivo study, osteoarthritis (OA) was induced in mice via DMM surgery, and the protective effects of PUE were evaluated. Micro‐CT imaging revealed that the DMM group exhibited pronounced joint space narrowing, osteophyte formation, and subchondral bone sclerosis. In contrast, PUE treatment (50 and 100 mg/kg) significantly reduced osteophyte size and improved bone volume/tissue volume (BV/TV), indicating a protective effect against structural degeneration (Figure 7A–D). Histological staining using H&E and Safranin O/Fast Green showed severe cartilage erosion and diminished matrix staining in the DMM group, whereas cartilage integrity was better preserved in PUE‐treated groups, accompanied by reduced OARSI scores and increased subchondral bone plate thickness (Figure 7E–H). Furthermore, immunohistochemical analysis demonstrated upregulation of Aggrecan expression in articular cartilage following PUE administration, indicative of enhanced matrix anabolism. TUNEL staining confirmed that PUE markedly decreased the number of apoptotic chondrocytes compared to the untreated DMM group (Figure 7I–L). Collectively, these results demonstrate that PUE effectively slows OA progression in vivo by preserving cartilage structure, reducing osteophyte burden, enhancing matrix protein expression, and inhibiting chondrocyte apoptosis, supporting its potential as a disease‐modifying agent for OA treatment.

## 4 Discussion

Osteoarthritis (OA) is a prevalent degenerative joint disease characterised by the progressive breakdown of articular cartilage, leading to pain, stiffness, and functional impairment in affected individuals [16]. This condition not only diminishes the quality of life for patients but also imposes a significant economic burden on healthcare systems worldwide due to increased medical costs and loss of productivity [17]. The multifactorial aetiology of OA encompasses various factors, including mechanical stress, genetic predisposition, inflammation, and metabolic dysregulation, contributing to the pathophysiological changes observed in the joint microenvironment [18]. Current management strategies primarily focus on symptom relief through analgesics and anti‐inflammatory medications, but these treatments fail to address the underlying disease mechanisms and progression of OA, highlighting the urgent need for novel therapeutic approaches that can modify the disease course and restore joint function [19, 20, 21].

In this context, our study investigates the therapeutic potential of PUE, a natural compound derived from the traditional Chinese herb *Pueraria lobata* , known for its anti‐inflammatory and chondroprotective properties. Utilising a comprehensive methodology that integrates bioinformatics analysis, molecular docking, and both in vitro and in vivo experiments, this research aims to elucidate the mechanisms by which PUE may exert its beneficial effects on chondrocytes and modulate inflammatory responses in OA. Our findings reveal that PUE not only inhibits pro‐inflammatory cytokine production but also protects against chondrocyte apoptosis and promotes ECM synthesis, thereby offering promising insights into its role as a disease‐modifying agent in the management of OA [22, 23].

The integration of bioinformatics and experimental validation in our study presents significant advances in understanding the therapeutic potential of PUE in osteoarthritis (OA). By identifying a total of 57 overlapping targets between PUE and OA‐related genes, we have established a multi‐target interaction model that suggests a nuanced approach to OA treatment. This is in alignment with the notion that complex diseases like OA often necessitate therapies that target multiple pathways simultaneously rather than relying on a single‐agent strategy. The biological processes enriched among these targets, particularly those related to collagen metabolism and ECM organisation, highlight the critical role of PUE in modulating the underlying pathophysiological mechanisms of OA [24]. Moreover, beyond the PI3K‐AKT pathway, some of these core targets—such as MMP9, which plays a key role in ECM degradation, and PTGS2, which mediates inflammatory responses—may interact with or influence the PI3K‐AKT axis. This potential crosstalk suggests that PUE's effects in OA could be mediated through a ‘multi‐target–multi‐pathway’ regulatory network, in which modulation of multiple interconnected pathways may produce synergistic benefits in reducing cartilage degeneration and inflammation. Future investigations should delve deeper into the specific roles of these overlapping targets to elucidate their contributions to OA pathogenesis and explore their potential as biomarkers for therapeutic efficacy.

Our findings regarding the cytotoxic effects of PUE at elevated concentrations elucidate the importance of establishing safe dosage ranges for therapeutic application. Notably, while concentrations of 5 and 10 μM were non‐cytotoxic and effective in promoting chondrocyte viability and function, higher concentrations resulted in decreased cell viability. This underlines the necessity of careful dose optimisation in clinical settings to maximise therapeutic benefits while minimising adverse effects [25]. Future studies should focus on the long‐term effects of PUE at these optimal concentrations and investigate the underlying mechanisms responsible for the observed cytotoxicity at higher doses. Such exploration can provide critical insights into the safe application of PUE in OA treatment, ensuring that it can be developed into a viable therapeutic option.

The protective effects of PUE against ECM degradation, evidenced by its ability to reverse IL‐1β‐induced declines in Aggrecan and Collagen II while inhibiting MMP‐13 and ADAMTS5 expression, further reinforce its therapeutic prospects. The preservation of cartilage integrity is paramount in OA management, and the ability of PUE to modulate ECM dynamics suggests its potential as a disease‐modifying agent [26]. Previous studies have indicated that the degradation of ECM components is closely linked to the progression of OA, making the modulation of these pathways a focal point for therapeutic interventions [27]. Future research should delve deeper into the molecular pathways through which PUE exerts these protective effects and consider its use in combination therapies that enhance ECM preservation.

Furthermore, our results demonstrate that PUE effectively mitigates IL‐1β‐induced chondrocyte apoptosis by regulating the intrinsic apoptotic pathway. The restoration of BCL‐2 levels and reduction of cleaved caspase‐3 expression highlight PUE's dual mechanism of action in protecting chondrocytes and maintaining cartilage matrix integrity. This finding aligns with existing literature that emphasises the significance of preventing chondrocyte apoptosis to delay OA progression [28]. Investigating the potential of PUE to be used in conjunction with other anti‐apoptotic therapies could pave the way for new combinatorial strategies that enhance its effectiveness in OA management.

Lastly, the modulation of inflammatory responses by PUE, as evidenced by the marked reduction in the expression of pro‐inflammatory cytokines, reinforces its role as an anti‐inflammatory agent. The ability of PUE to suppress IL‐1β‐induced inflammatory mediators positions it as a promising candidate for addressing the chronic inflammation associated with OA. Compared with conventional OA therapeutic agents such as NSAIDs and chondroprotective agents, which often act through single targets and may carry gastrointestinal or cardiovascular risks, PUE's multi‐target and multi‐pathway modulation offers potential advantages in both efficacy and safety. Additionally, its dose‐dependent effects and potential for combination therapy provide a novel therapeutic perspective that addresses some limitations of existing single‐target treatments. Future studies should explore the broader implications of PUE's anti‐inflammatory effects and their potential applicability in other inflammatory conditions, which may provide valuable insights into developing comprehensive therapeutic regimens for OA and related disorders.

This study is not without limitations, which warrant careful consideration when interpreting the findings. Firstly, the lack of clinical validation restricts the direct applicability of the results to human osteoarthritis, necessitating further research to establish the therapeutic efficacy of PUE in clinical settings. Additionally, the relatively small sample size utilised in the animal models may hinder the generalisability of our results. The absence of comprehensive in vivo mechanistic studies limits our understanding of the multifaceted effects of PUE on cartilage health and inflammatory pathways. Future research should address these gaps by incorporating larger sample sizes and exploring the intricate molecular mechanisms involved in PUE's actions within a clinical context.

In conclusion, our findings provide compelling evidence that PUE exerts protective effects against chondrocyte apoptosis and ECM degradation while modulating inflammatory responses in osteoarthritis. The multifaceted actions of PUE highlight its potential as a disease‐modifying agent, suggesting that it may serve as a promising therapeutic option to address the underlying progression of OA. These insights pave the way for future clinical applications and underscore the importance of continued research to fully elucidate the therapeutic benefits of PUE in managing osteoarthritis.

## Author Contributions

**Yanjing Zhang:** conceptualization (equal), writing – original draft (equal), writing – review and editing (equal). **Zhiqiang Chen:** conceptualization (equal), writing – original draft (equal). **Ye Cheng:** conceptualization (equal), software (equal), validation (equal). **Yuxuan Zhou:** conceptualization (equal), methodology (equal). **Yaoheng Yang:** conceptualization (equal), software (equal). **Mengjiao Che:** conceptualization (equal). **Yiwen Zhang:** conceptualization (equal), writing – review and editing (equal). **Yalan Li:** conceptualization (equal), writing – original draft (equal), writing – review and editing (equal).

## Ethics Statement

All animal experiments were conducted following the guidelines set forth by the Institutional Animal Care and Use Committee (IACUC) of the Third Affiliated Hospital of Southern Medical University (Approval No: TOP‐IACUC‐2023‐0333). The study adhered to all applicable national and international laws regarding animal welfare, and the methods were designed to minimise animal suffering. This research was conducted in compliance with the ARRIVE 2.0 guidelines, ensuring rigorous ethical and scientific standards.

## Conflicts of Interest

The authors declare no conflicts of interest.

## Data Availability

The datasets used and/or analysed during the current study are available from the corresponding author upon reasonable request.
